# Ion Transport Regulation by TRPV4 and TRPV1 in Lens and Ciliary Epithelium

**DOI:** 10.3389/fphys.2021.834916

**Published:** 2022-01-31

**Authors:** Nicholas A. Delamere, Mohammad Shahidullah

**Affiliations:** Department of Physiology, University of Arizona, Tucson, AZ, United States

**Keywords:** TRPV4, TRPV1, Na, K-ATPase activity, NKCC1, lens epithelium, ciliary epithelium

## Abstract

Aside from a monolayer of epithelium at the anterior surface, the lens is formed by tightly compressed multilayers of fiber cells, most of which are highly differentiated and have a limited capacity for ion transport. Only the anterior monolayer of epithelial cells has high Na, K-ATPase activity. Because the cells are extensively coupled, the lens resembles a syncytium and sodium-potassium homeostasis of the entire structure is largely dependent on ion transport by the epithelium. Here we describe recent studies that suggest TRPV4 and TRPV1 ion channels activate signaling pathways that play an important role in matching epithelial ion transport activity with needs of the lens cell mass. A TRPV4 feedback loop senses swelling in the fiber mass and increases Na, K-ATPase activity to compensate. TRPV4 channel activation in the epithelium triggers opening of connexin hemichannels, allowing the release of ATP that stimulates purinergic receptors in the epithelium and results in the activation of Src family tyrosine kinases (SFKs) and SFK-dependent increase of Na, K-ATPase activity. A separate TRPV1 feedback loop senses shrinkage in the fiber mass and increases NKCC1 activity to compensate. TRPV1 activation causes calcium-dependent activation of a signaling cascade in the lens epithelium that involves PI3 kinase, ERK, Akt and WNK. TRPV4 and TRPV1 channels are also evident in the ciliary body where Na, K-ATPase is localized on one side of a bilayer in which two different cell types, non-pigmented and pigmented ciliary epithelium, function in a coordinated manner to secrete aqueous humor. TRPV4 and TRPV1 may have a role in maintenance of cell volume homeostasis as ions and water move through the bilayer.

## Introduction

The lens is a deceptively simple tissue, but its transparency and optical properties are the result of many complex cellular specializations. Aside from a monolayer of epithelium at the anterior surface, it is formed by a vast number of almost identical fiber cells arranged with geometric precision in a hexagonal array. There are no blood vessels, no nerves. The lens fiber cells are differentiated to the extent that most fibers have no nucleus, mitochondria or endoplasmic reticulum. Absence of these particles minimizes light scattering, which improves lens transparency. A particularly unusual feature of lens fibers is their lack of Na, K-ATPase activity. Most of the cells in the lens are poorly equipped for primary active sodium-potassium transport. Only the anterior monolayer of epithelial cells has high Na, K-ATPase specific activity (Tamiya et al., 2003; Delamere and Tamiya, 2004). Because lens cells are extensively coupled, the entire structure resembles a syncytium. Here we describe recent studies that suggest TRPV4 and TRPV1 ion channels activate signaling pathways that play an important role in matching epithelial ion transport activity with needs of the lens cell mass.

## TRPV4 – Na, K-ATPase Signaling Cascade

Na, K-ATPase-mediated active transport is considered essential in almost all mammalian cells. There is considerable interest in understanding how the lens achieves ion homeostasis in a multicellular structure in which most cells have negligible Na, K-ATPase activity (Delamere and Dean, 1993). Lens water homeostasis, and ultimately refractive index and transparency, depend on maintenance of steady state cytoplasmic sodium and potassium concentrations. Age-related human cortical cataract is frequently associated with abnormal sodium and potassium balance in the lens. It has been known for a long while that the degree of lens opacification observed in cortical cataracts aligns with the magnitude of an increase in lens sodium and decrease in potassium (Duncan and Bushell, 1975). Moreover, increases in lens sodium are generally accompanied by an increase in calcium which worsens transparency loss due to activation of calpains, crystallin protein aggregation, and possibly calcium precipitates (Li et al., 2021). In many cataractous lenses, Na, K-ATPase activity was found to be lower than normal (Kobatashi et al., 1982; Garner and Spector, 1986).

The specific activity of Na, K-ATPase is moderately high in the center of the epithelium and highest at the periphery of the monolayer. The center of the epithelium covers the anterior pole of the lens, while the periphery of the epithelium covers the equator of the lens. The newly formed, elongating, fibers at the lens equator have appreciable Na, K-ATPase specific activity, though this is only ∼ 20% of the specific activity in the epithelium. Elsewhere in the fiber mass, Na, K-ATPase specific activity ranges from 4 – 0.2% of that in the epithelium (Delamere and Dean, 1993; Tamiya et al., 2003; Delamere and Tamiya, 2004). As mentioned later, the asymmetrical distribution of Na, K-ATPase activity in the epithelium and fibers gives rise to ionic currents that flow in and around the lens. It is interesting that in spite of their low Na, K-ATPase activity, lens fibers have abundant Na, K-ATPase catalytic (α) subunit protein (Delamere and Dean, 1993). This could be due in part to retention of old, inactive, Na, K-ATPase protein. In addition, tyrosine phosphorylation of the α subunit protein reduces Na, K-ATPase activity in fibers (Bozulic et al., 2004).

Because the lens fibers have inadequate Na, K-ATPase activity, sodium and potassium homeostasis of the entire cell structure is largely dependent on Na, K-ATPase activity in epithelial cells even though this monolayer of undifferentiated epithelial cells makes up a miniscule fraction of the lens (Gao et al., 2000). For obvious reasons, regulation of Na, K-ATPase activity in the epithelium became a topic of interest. In previous studies, Duncan and coworkers demonstrated functional purinergic receptor responses in the lens epithelium (Collison et al., 2000; Duncan and Collison, 2003) but, at that time, the functional significance of purinergic receptors was unclear. Later, we discovered changes in lens Na, K-ATPase activity in the epithelium of lenses exposed to purinergic agonists. We also demonstrated a possible role for tyrosine kinase signaling in the chain of events that regulates Na, K-ATPase activity (Okafor et al., 1999; Okafor and Delamere, 2001; Tamiya et al., 2007). The stimulation of Na, K-ATPase activity in the epithelium of lenses exposed to purinergic agonists was found to depend on Src family tyrosine kinase activation (Tamiya et al., 2007).

**FIGURE 1:**
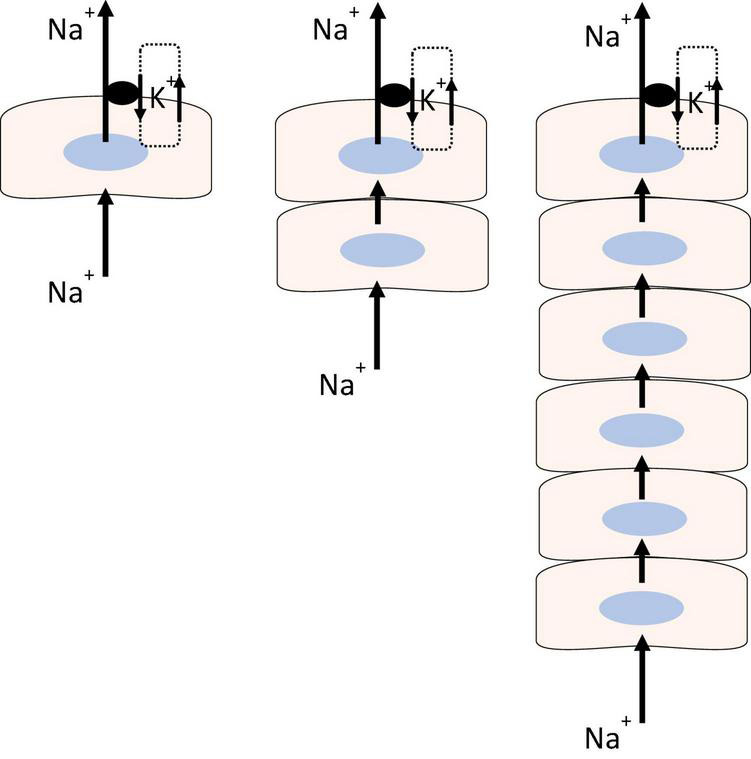
Sodium-potassium homeostasis in a single cell (Left), is made possible because Na, K-ATPase activity increases or decreases in response to changes in cytoplasmic sodium concentration at the sodium ion binding site. The model shows an arrangement in which the cell with Na, K-ATPase activity is required to maintain steady state sodium and potassium concentrations in two (Center) or more coupled cells (Right) that have little or no Na, K-ATPase activity. The model depicts sodium ions flowing through the coupled cells to be actively transported outward by Na, K-ATPase while potassium channels recirculate potassium ions the Na, K-ATPase brings into the cell.

**FIGURE 2:**
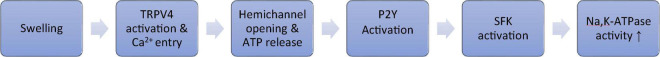
Swelling activates TRPV4. Schematic diagram of the TRPV4-initiated signaling cascade that culminates in an increase of Na, K-ATPase activity.

All cells are required to compensate for osmotic swelling and shrinkage that may arise under a variety of physiological and pathophysiological conditions (McManus et al., 1995; Strange, 2004). With few exceptions, this requires Na, K-ATPase. Active transport by Na, K-ATPase establishes and maintains sodium and potassium ion concentration gradients across the plasma membrane and consequently plays a critical role in determining membrane potential, cytoplasmic chloride ion concentration, and cell volume (Armstrong, 2003). As long as sufficient ATP is available, the rate of Na, K-ATPase-mediated transport increases when cytoplasmic sodium concentration rises in the vicinity of the sodium binding site. This may work well enough to maintain steady state sodium and potassium ion concentrations in a single cell but the situation is more complicated in a syncytium of many coupled lens cells, most of which have no Na, K-ATPase activity. In respect to coupling and Na, K-ATPase distribution, lens tissue is similar to the multicell model shown in Figure 1 (right panel). Sodium and potassium homeostasis of the fiber mass is driven largely by Na, K-ATPase activity in epithelial cells (Gao et al., 2000) but the sodium binding site of Na, K-ATPase in the epithelium is not directly affected by changes of sodium ion concentration in remotely located fibers. We have evidence that Na, K-ATPase activity in the epithelium is regulated by a remote control mechanism using TRPV4 channels as sensors that respond to lens swelling (Shahidullah et al., 2015). TRPV4 is expressed in the lens. In the porcine lens TRPV4 protein is mainly in the epithelium while in mouse lens it is also evident in the fiber mass (Shahidullah et al., 2012b,^2015^; Nakazawa et al., 2019). Our studies in porcine lens suggest that when the lens is subjected to osmotic swelling, TRPV4 channels in the epithelium are activated to open by the mechanical stimulus. This permits Ca^2+^ entry and triggers a complex chain of signaling events that stimulates Na, K-ATPase activity (Figure 2) (Shahidullah et al., 2012b).

TRPV4 channel activation in the epithelium triggers calcium-dependent opening of connexin hemichannels (Shahidullah et al., 2012b; Mandal et al., 2015). The critical role of connexin hemichannels is illustrated by the observation that TRPV4-dependent responses are absent in lenses from Cx50 knockout mice (Delamere et al., 2020). However, there is some evidence to suggest TRPV4-dependent pannexin channel activation also occurs in response to a hypoosmotic stimulus (Shahidullah et al., 2012b). Either way, the channels that open permit ATP release from the lens. This stimulates purinergic receptors in the epithelium and results in the activation of Src family tyrosine kinases (SFKs) and SFK-dependent increase of Na, K-ATPase activity (Figure 2; Shahidullah et al., 2012a,b). TRPV4 channels are mechanosensitive (O’Conor et al., 2014) and it is reasonable to assume they are opened by stretching forces associated with osmotic swelling of the lens. Accordingly, the lens epithelium was found to exhibit a TRPV4-dependent response triggered by damage to fiber cells far from the anterior surface. The response, which involved rapid (1 min) activation of SFKs and an increase of Na, K-ATPase activity in the epithelium, could be abolished by TRPV4 inhibition with HC067047 (Shahidullah et al., 2015).

## TRPV1 – NKCC1 Signaling Cascade

**FIGURE 3:**
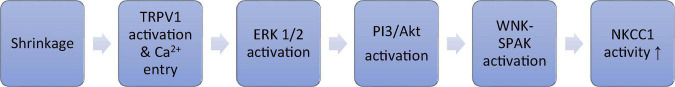
Shrinkage activates TRPV1. Schematic diagram of the TRPV1-initiated signaling cascade that culminates in an increase of NKCC1 activity.

While TRPV4 is critical to the lens response to osmotic swelling, a separate, opposing, signaling cascade is activated by TRPV1 and corrects for lens shrinkage. The TRPV1 loop stimulates the activity of a different ion transporter, the Na/K/2Cl cotransporter NKCC1 (Gao et al., 2015; Mandal et al., 2018; Shahidullah et al., 2018). Various lines of evidence suggest the TRPV1 mechanism increases NKCC1 activity in the epithelium when the lens is exposed to a hyperosmotic stimulus (Shahidullah et al., 2018, 2020). The TRPV1 agonist capsaicin and hyperosmotic stimuli similarly increase the rate of NKCC-mediated ion transport as determined experimentally by measuring bumetanide-sensitive Rb uptake (Shahidullah et al., 2018). TRPV1 activation causes calcium-dependent activation of a signaling cascade in the lens epithelium that involves PI3 kinase, ERK, Akt and WNK (Figure 3; Shahidullah et al., 2018). This leads to increased NKCC1 activity which, by loading solute into the cells, has the effect of reversing osmotic shrinkage. There is rich expression of NKCC1 and TRPV1 in human and pig lens epithelium (Chee et al., 2010; Mandal et al., 2018; Shahidullah et al., 2018). Quantitative RT-PCR studies showed that TRPV1 mRNA abundance was the highest in the lens among the ocular tissues (Martínez-García et al., 2013). In an elaborate study using western blot and immunohistochemistry, expression of TRPV1 was evident in both epithelium and fiber cells of the mouse lens (Nakazawa et al., 2019). Different regions of the fiber mass displayed differences in subcellular distribution of TRPV1 protein between plasma membrane. Finding expression of TRPV1 in the lens was unanticipated. TRPV1 is better known for its neuronal role in pain sensation and as the receptor for capsaicin. Interestingly, TRPV1 in neurons is activated by compressive forces caused by cell shrinkage (Prager-Khoutorsky et al., 2014).

## Lens Circulation

**FIGURE 4:**
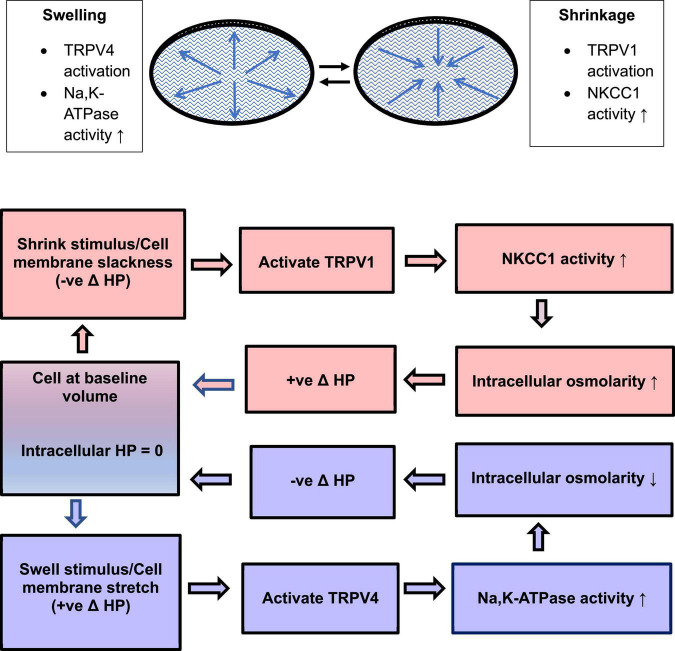
To maintain a steady state, the lens may sense and respond to minor episodes of swelling and shrinkage by means of a TRPV4 mechanism that regulates Na, K-ATPase activity and a TRPV1 mechanism that regulates NKCC1 activity. TRPV4 and TRPV1 are activated by increases or decreases of intracellular hydrostatic pressure (HP) in lens surface cells. The feedback loops return HP to zero.

The extensive coupling of lens cells, together with the regional separation of Na, K-ATPase activity to the equatorial surface of the lens, and the sodium ion leak conductance of fiber cells, gives rise to a circulatory flow of sodium ions through the packed cell structure (Donaldson et al., 2001). The circulatory flow of sodium creates small osmotic gradients that cause water to move inward at the anterior and posterior poles and outward at the lens equator (Mathias et al., 2007; Vaghefi et al., 2011; Candia et al., 2012). It is a matter of significance that the circulatory flow also contributes to a gradient of water content in the fiber mass (low in the center) which in turn may help establish a gradient of refractive index that impacts focusing power and corrects spherical aberration (Donaldson et al., 2017). As might be expected, the refractive index gradient is disrupted by the Na, K-ATPase inhibitor ouabain (Vaghefi et al., 2015). The circulatory flow of water creates a parabolic gradient of intracellular hydrostatic pressure (HP), highest at the center of the lens and zero at the surface cells (Mathias et al., 2007; Gao et al., 2011, 2013). Because the magnitude of HP in the lens cortex is dependent on the activity of the ion transporters that drive the circulatory flow of ions and water, it is no surprise that hydrostatic pressure responses in surface cells of intact lenses indicate the functional importance of TRPV4 and TRPV1. In a series of painstaking studies on intact mouse lenses, Mathias and coworkers demonstrated that TRPV4 and TRPV1 channels are the sensors in two different feedback loops that respond to changes of HP in lens surface cells (Gao et al., 2015; Delamere et al., 2020; Shahidullah et al., 2020). One feedback loop uses TRPV4 as a sensor that detects a positive change in HP and corrects for it by stimulating Na, K-ATPase activity to reduce intracellular osmolarity and return HP to zero. The other feedback loop uses TRPV1 as a sensor that detects a negative change in HP and corrects for it by stimulating NKCC1 to increase intracellular osmolarity and restore the HP to zero. Under normal conditions, the lens may alternate between episodes of swelling or shrinkage that are detected and brought back to baseline by the TRPV4 or TRPV1 feedback loop mechanisms (Figure 4).

## Ciliary Epithelium

**FIGURE 5:**
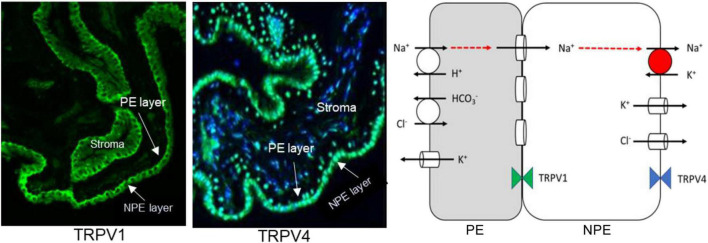
Expression of TRPV1 (Left Panel) and TRPV4 (Middle Panel) in porcine ciliary epithelium. Immunolocalization was carried out on paraffin sections as described previously (Shahidullah and Delamere, 2014). The scheme on the right depicts the localization of transport mechanisms that contribute to transepithelial solute and water movement that supports aqueous humor formation. It is adapted from Charles W. McLaughlin et al. (McLaughlin et al., 2004). The model has been simplified for clarity. Aqueous humor flow occurs in the PE-to-NPE direction. The PE and NPE are coupled by gap junctions and function in the manner of a syncytium. Na, K-ATPase activity is highest at the basolateral, aqueous humor-facing, surface of the NPE. In respect to coupling and Na, K-ATPase distribution, the tissue is similar to the two-cell model shown in Figure 1.

The functional role of TRPV4 and TRPV1 in the lens led us to examine the ciliary epithelium which is a bilayer structure formed by two different epithelial cell types in which one cell type, the non-pigmented ciliary epithelium (NPE), is specialized for Na, K-ATPase activity (Riley and Kishida, 1986; Usukura et al., 1988; Ghosh et al., 1991). The apical surfaces of the pigmented ciliary epithelium (PE) and NPE face each other and the two cells are efficiently coupled by gap junctions (Civan and Macknight, 2004). Most Na, K-ATPase activity is localized to the basolateral aqueous humor-facing surface of the NPE (Usukura et al., 1988; Ghosh et al., 1991). In terms of coupling and distribution of Na, K-ATPase activity, the ciliary epithelium bilayer can be simplified to the two-cell model shown in Figure 1 (middle panel). In porcine eyes, TRPV4 was detected in the NPE cells, predominantly but not exclusively on the basolateral surface (Figure 5; Delamere et al., 2016). Interestingly, rich expression of connexin-50 was also detected on the basolateral surface of NPE (Shahidullah and Delamere, 2014). The unpaired connexin-50 suggests TRPV4 may have role in regulation of hemichannel opening. Of note, pannexin-1 (Panx1) is also detectable at the same location. It was interesting to also discover TRPV1 in the ciliary epithelium bilayer (Figure 5, left). TRPV1 was most evident at the junction between NPE and PE.

The functional significance of TRPV4 and TRPV1 channels in the ciliary epithelium remains to be determined. The NPE and PE work in a coordinated manner to form aqueous humor. The two cell types express different transport mechanisms. PE but not NPE cells display a regulatory volume increase when subjected to osmotic shrinkage (Edelman et al., 1994). In contrast, NPE but not NPE cells display a regulatory volume decrease when subjected to osmotic swelling (Edelman et al., 1994). The PE appears specialized for solute entry while the NPE is specialized for solute exit in terms of transporter expression (Edelman et al., 1995; Walker et al., 1999). Moreover, the NPE has an elaborately folded basolateral plasma membrane characteristic of a secretory cell (Usukura et al., 1988). Solute and water move through the bilayer, and Brubaker calculated that a ciliary epithelial cell transports the equivalent of 30% of its own volume every minute (Brubaker, 1991). It follows that the cells are at risk of swelling or shrinkage whenever there is a slightest mismatch between entry and exit of water that flows through them. In corneal endothelium as well as ciliary epithelium, it has been suggested that transcellular fluid transport could be associated with cell volume oscillation as water enters and exits in a pulsatile manner (Fischbarg, 1997; Walker et al., 1999). It is possible that TRPV4 and TRPV1 have a role in maintenance of cell volume homeostasis in the bilayer. Jo and coworkers (Jo et al., 2016) reported swelling-induced calcium responses in NPE but not PE. The same study showed TRPV4 inhibition prevented the swelling-induced calcium responses in mouse NPE and absence of the responses in TRPV4 knockout mice. Because the eye is a closed structure, the balance between aqueous humor formation by the ciliary epithelium and exit through the trabecular meshwork and uveoscleral outflow pathways gives rise to intraocular pressure (IOP). Pharmacological strategies to lower IOP, sought after for glaucoma therapy, include carbonic anhydrase inhibitors and adrenergic drugs that reduce production of aqueous humor, as well as cholinergic agents, prostaglandins and Rho kinase inhibitors that increase aqueous outflow (Schmidl et al., 2015; Rao et al., 2017; Marshall et al., 2018). It has been reported that the TRPV4 agonist GSK1016790A reduces IOP and TRPV4 knockout mice have elevated IOP (Luo et al., 2014). However, others have reported IOP is unchanged in TRPV4 knockouts (Ryskamp et al., 2016). Interpretation of IOP responses is far from straightforward because TRPV4 may influence both formation and drainage of fluid. It is suggested that TRPV4 has a role in regulating function of the trabecular meshwork and intraocular pressure and Schlemm’s Canal structures that constitute the aqueous humor outflow tract (Yarishkin et al., 2019; Lakk and Križaj, 2021; Patel et al., 2021). In studies on the different IOP responses to systemic and topical administration of a TRPV4 antagonist HC067047, the functional link between TRPV4 and the primary cilium in trabecular meshwork cells has been considered important (Luo et al., 2014; Ryskamp et al., 2016).

## Discussion

Several lines of evidence illustrate the calcium dependence of lens TRPV1- and TRPV4-mediated responses to osmotic stimuli (Shahidullah et al., 2012b; Mandal et al., 2015). Responses do not occur when calcium is omitted from the bathing medium, which suggests that even though TRPV1 and TRPV4 are non-selective cation channels (White et al., 2016), their functional role appears to be calcium entry. An important question that remains unanswered is why the two channels have such different calcium signaling-dependent effects on cell function. TRPV1 activation leads to stimulation of NKCC1 while TRPV4 activation leads to stimulation of Na, K-ATPase.

TRPV1, TRPV4, and other TRP channels are polymodally regulated (Nilius and Szallasi, 2014). TRPV1, for example, is activated by temperature and a range of chemical stimuli. The focus here is on TRPV1 activation by the mechanical forces associated with cell shrinkage and TRPV4 activation by stretching forces associated with cell swelling. There is more to be learned regarding precise mechanism of channel activation by mechanical forces. It has been suggested that TRP channels themselves may not necessarily be sensitive to deformation of the lipid bilayer, being activated instead by cytoskeletal tethers or diffusible molecules that result from phospholipase A2 activation (Everaerts et al., 2010). There may even be circumstances in which a different channel, Piezo1, detects the mechanical stimulus and TRPV4 channel activation occurs downstream of Piezo1 activation (Swain et al., 2020; Swain and Liddle, 2021). It is noteworthy that Piezo1 is expressed in the NPE cell layer of the ciliary body (Fang et al., 2021; Zhu et al., 2021) as well as TM cells in the aqueous outflow pathway (Yarishkin et al., 2021; Zhu et al., 2021).

Cell volume homeostasis requires the ability to respond to episodes of swelling or shrinkage and correct volume back to baseline by activating various ion channels and transporters. TRPV4 or TRPV1 appear to function as sensors in feedback loop mechanisms that adjust the activity of Na, K-ATPase and NKCC1. Details on the precise mechanism of channel activation have yet to emerge but TRPV4 and TRPV1 certainly respond to mechanical stimuli, evident in studies on responses to stretching or relaxing tension on the lens (Chen et al., 2019). Recent works suggests the channels are exquisitely selective. Osmotic swelling does not activate the TRPV1 feedback pathway nor does osmotic shrinkage activate the TRPV4 feedback pathway. Further study will be needed to explain how TRPV4 apparently responds to stretch while TRPV1 responds to shrinkage. When the channels are connected to the appropriate signaling pathway, Na, K-ATPase and NKCC1 effectively become mechanosensitive. This may be an important aspect of ion and water homeostasis in coupled cell structures like the lens and ciliary epithelium.

## Author Contributions

Both authors listed have made a substantial, direct, and intellectual contribution to the work, and approved it for publication.

## Conflict of Interest

The authors declare that the research was conducted in the absence of any commercial or financial relationships that could be construed as a potential conflict of interest.

## Publisher’s Note

All claims expressed in this article are solely those of the authors and do not necessarily represent those of their affiliated organizations, or those of the publisher, the editors and the reviewers. Any product that may be evaluated in this article, or claim that may be made by its manufacturer, is not guaranteed or endorsed by the publisher.
